# The Effect of Females’ Reproductive Factors on Pituitary Gland Size in Women at Reproductive Age

**DOI:** 10.3390/medicina55070367

**Published:** 2019-07-11

**Authors:** Mohammad Hossein Daghighi, Fatemeh Seifar, Alireza Parviz, Masoud Poureisa, Farid Hajibonabi, Shadi Daghighi, Rosa Golshan

**Affiliations:** 1 Department of Radiology, Tabriz University of Medical Sciences, Tabriz 5166/15731, Iran; 2 Stem Cell Research Center, Aging Research Institute, Faculty of Medicine, Tabriz University of Medical Sciences, Tabriz 5166/15731, Iran; 3 Student Research Committee, Tabriz University of Medical Sciences, Tabriz 5166/15731, Iran; 4 Faculty of Medicine, Tabriz University of Medical Sciences, Tabriz 5166/15731, Iran

**Keywords:** pituitary hormones, estrogen, gravidities, parity, postpartum period, neuroimaging

## Abstract

*Background and Objectives*: The brain imaging of the pituitary gland in females has shown a change in pituitary size and volume in the female’s population. It has been proven that the pituitary gland is affected by pregnancy, giving birth, and hormone-related factors. Therefore, this study aimed to evaluate the factors which may have an impact on the pituitary size in females at reproductive age and compare the pituitary size in females with a history of pregnancy, those at the postpartum period, and nullipara females. *Materials and Methods*: This population-based study was conducted on 208 healthy women aged 12–55 years old. Participants underwent cranial Magnetic resonance imaging (MRI), and pituitary diameters (craniocaudal, anteroposterior, and transverse) and volume were measured for each subject. The correlation of age, gravity, parity, lactation, and intake of oral contraceptives with pituitary size were analyzed. *Results*: One-hundred and eighty females met the criteria for participation. The pituitary volume correlated negatively with hormone-related factors. The gravity (*r* = −0.35) and parity (*r* = −0.35) had significant negative effects on the pituitary volume (*p* < 0.001). The use of oral contraceptives and lactation were also in negative correlation with the pituitary volume (*r* = −0.20, *p* = 0.006, *r* = −0.56, *p* < 0.001, respectively). The craniocaudal diameter was also affected by gravity (*r* = −0.62), parity (*r* = −0.57), intake of contraceptives (*r* = −0.32), and lactation (*r* = −0.70), *p* < 0.001. The anteroposterior diameter of the pituitary gland associated significantly with gravity (*r* = −0.19, *p* = 0.009), parity (*r* = −0.20, *p* = 0.007), and lactation (*r* = −0.25, *p* = 0.001). The transverse diameter of the pituitary gland also related negatively with reproductive factors such as gravity (*r* = −0.15, *p* = 0.04), parity (*r* = −0.17, *p* = 0.02), and lactation (*r* = −0.17, *p* = 0.02). The pituitary gland of nullipara females was the greatest in size. Recent pregnancy led to increased craniocaudal and anteroposterior diameters. *Conclusions*: In this study, we found a negative effect of pregnancy and giving birth on pituitary size. Nullipara females were found to have the greatest pituitaries, even greater than the females in the postpartum period.

## 1. Introduction

In cranial magnetic resonance imaging (MRI), the pituitary gland of women is greater in size compared to men [1]. This may be due to the hypertrophy and hyperplasia of lactotroph cells of the anterior pituitary gland during pregnancy. Pregnancy is associated with some pituitary disorders such as Sheehan’s syndrome, prolactinoma, Cushing’s disease, and lymphocytic hypophysitis, which can affect the size of the pituitary gland in female subjects [2].

In some cases, physiological enlargement of the gland can be misdiagnosed with a hypophysis adenoma [3]. In this context, reduced size of the pituitary gland is presented in Sheehan’s syndrome [2].

The association between age, gender, and hormonal factors has been proved previously. The majority of the former studies have described the change in the gland size and the function in the normal healthy population or different age groups of female subjects [4,5,6,7]. It is worth to mention that the main culprits of pituitary size change are hormonal changes during pregnancy. During pregnancy, there is an elevation in adrenocorticotropic hormone (ACTH) and prolactin (PRL) level and a decrease in Follicle-stimulating hormone (FSH), Luteinizing hormone (LH), and thyroid stimulating-hormone (TSH) levels due to increased placental Estrogen and Progesterone hormones [2,8,9]. It is also suggested that pituitary gland retains its normal shape and size within six months after giving birth [10]; however, in some studies, there was still an enlargement in the pituitary size after giving birth [9]. Our hypothesis is that in females who have normal hormonal levels, the pituitary size may have already been affected by reproductive factors, such as pregnancy, giving birth, lactation, and the use of external hormones.

Thus, the association of gland size and volume with reproduction-related factors such as parity, gravity, lactation, and intake of oral contraceptives in women during reproductive age is a matter of concern when evaluating cranial magnetic resonance (MR) images of young and middle-aged women.

Studies have shown an enlargement in the size of the pituitary during pregnancy and a deterioration in its size after the postpartum phase of giving birth [2]. It is expected that the pituitary gland would return to its normal size after giving birth. However, the normal size of the gland in the postpartum period, in females who have given birth to a child, and in nulligravid females has not been studied.

We hypothesized that pituitary size in healthy women is affected by their reproductive factors. It seems important to know the possible physiologic factors that change the pituitary size and to what degree a normal pituitary gland may be changed by these factors. This will help radiologists to avoid any misunderstanding of the physiologic enlargement/shrinkage of the pituitary with other pathologic features of the gland.

Therefore, this study aimed to evaluate the factors which may have an impact on the pituitary size in females at reproductive age and compare the mean pituitary size in females who have had a history of pregnancy with those who have not.

## 2. Materials and Methods

This population-based cross-sectional study was conducted on two-hundred and eight women between March 2017 and September 2018 at the Radiology Department of Imam Reza Hospital, Tabriz, Iran. The included study subjects were females at reproductive age (12–55 years old) who were admitted for a brain MRI evaluation of various diseases and met the inclusion criteria. The participants’ medical records were assessed for the conditions listed as the exclusion criteria (see section below). The participants were also asked to fill a questionnaire about their age, marital status, number of pregnancies, and childbearing, the occurrence of postpartum bleeding, the time since the last childbearing and lactation, and the use of oral contraceptives (OCP). For OCP intake, females were those who were using OCP unceasingly from 1 to 20 months (5.66 ± 4.6 months) at least until a month prior to the imaging.

### 2.1. Exclusion Criteria

The participants with the following conditions were excluded from further evaluation: females with a history of brain surgery and any previous pituitary diseases, subjects with hormonal disorders including adrenal diseases (e.g., Cushing’s diseases), thyroid diseases (hypo/hyperthyroidism), gonadal diseases, past medical history of psychiatric disorder, present use of antipsychotic or anticonvulsants medications, subjects with abnormal MR findings such as hydrocephalus, high intracranial pressure, and pituitary adenoma. The sexual hormones were also recorded in some of the patients’ records. Those females who were suspected of having a malfunction in their hypothalamus-pituitary-ovarian pathway. Among these females, we only included females with normal levels of PRL, FSH, LH, and TSH hormones.

### 2.2. Ethical Considerations

The study protocol was approved by the Local Ethics Committee of Tabriz University of Medical Sciences (IR.TBZMED.REC.1395.1063; date: 2017.14.01.). All the participants were provided with complete and instructive information about the project and written informed consent was received from each of them.

### 2.3. Imaging Procedure

The prospective cranial MRI images were obtained using a 1.5 T1-weighted turbo spin-echo (TSE) sequence (TR: 2800 msec, TE: 100 msec, slices: 2.5 mm, inter-slice gap: 0.3 mm, repetition: 1, voxel volume: 1 mm^3^, mutation angle: 90°, Field-of-view (FOV): 220 mm, duration of scanning: 35 min). MRI scanning was performed with a Tesla Avanto, Siemens system (Erlangen, Germany) at the Department of Radiology of Imam Reza University-Hospital (Tabriz, Iran).

Subjects were aligned supine along the midline in the scanner with head support used to reduce the potential motion artifacts by immobilization. For the volumetric analysis, the sagittal, axial and coronal views were obtained. The size of the pituitary gland was measured by PC workstation using the Image-J software [11] with standard-setting.

### 2.4. Measurement of the Pituitary Dimensions

**Figure 1:**
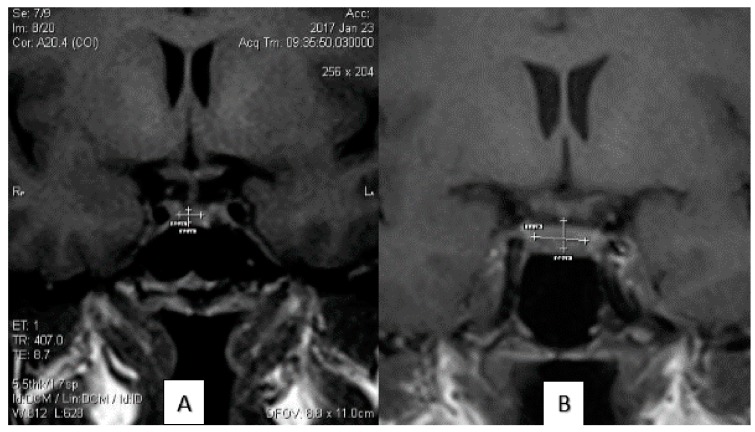

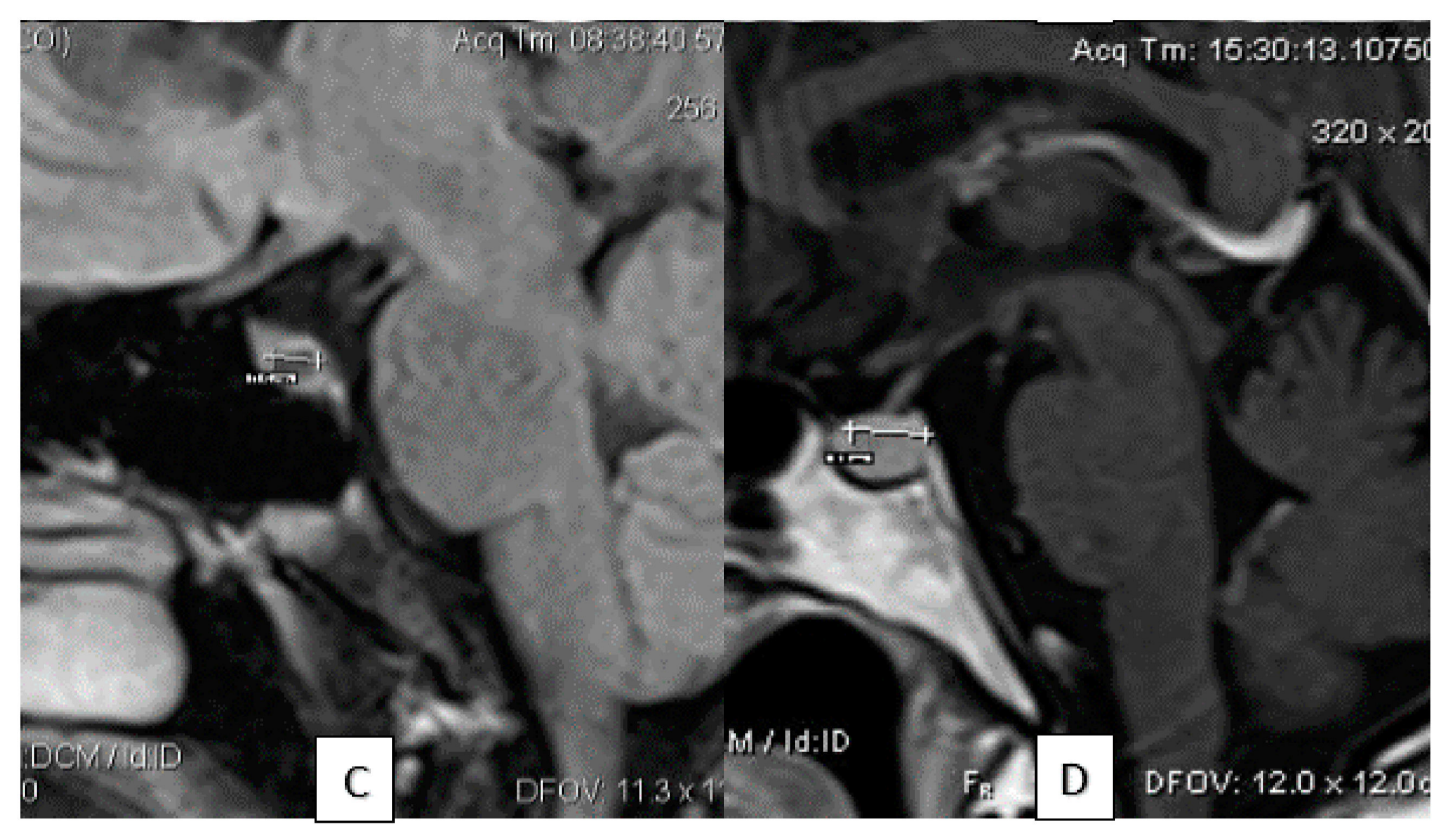
(**A**) Forty-one year old multiparous female with a history of intake of oral contraceptives; (**B**) Thirty-five year old multiparous female, negative for the use of oral contraceptives; (**C**) twenty-five year old nulliparous female, negative for the use of oral contraceptives; (**D**) forty-eight year old multigravida nulliparous female, negative for the use of oral contraceptives. (**A**) and (**B**) Coronal view. Transverse and Craniocaudal Diameters. (**C**) and (**D**) Sagittal view. Anteroposterior Diameters.

The calculation of the size of the pituitary gland was performed by two professional radiologists (M.H.D. and M.P.) with at least 10 years of experience in MRI reporting. The data were double-checked by a senior resident of radiology. The height of the pituitary gland was analyzed on the mid-sagittal view on the workstation. The craniocaudal diameter (height) of the pituitary gland was determined from the upper border at the site of insertion of the stalk to the lower border of the gland considering the maximum vertical diameter. The anteroposterior (length) diameter of the gland was calculated in the horizontal plane. The gland’s width was determined in the coronal view as the maximum transverse diameter of the gland. The pituitary volume was measured by Image-J software via a semiautomatic voxel-based analysis. The measurement of each diameter MRI images is delineated in Figure 1.

### 2.5. Statistical Analysis

The patients’ characteristic data and the MRI measured variables were recorded for statistical analysis by the SPSS software version 19.0 (IBM Corp., Armonk, NY, USA). Each diameter of the gland was reported as the mean ± standard deviation (SD). The Spearman correlation coefficient was applied to assess the possible correlation between pituitary size and giving birth, use of oral contraceptives, pregnancies and lactation. To compare the mean size of the gland in each productive stage (e.g., nullipara and those who have given birth), an independent samples *t*-test was used. A multivariate regression analysis was applied to correct the results for age and to find the dominant factor. A *p* < 0.05 was considered significant. The graphs were depicted by Graphpad Software version 6.0 (Graphpad PRISM Inc., San Diego, CA, USA).

## 3. Results

### 3.1. General Findings

During eighteen months of study, 208 females met the inclusion criteria and their brain MR images were evaluated. Out of them, nine were excluded for pathological MR findings (seven pituitary adenoma, two hydrocephalous) and 19 for incomplete data acquisition.

The indications of the MR imaging is shown in Table 1. Headache and vertigo were the most common complaints of the imaging indications.

One-hundred and seventy-four of the females were married, 124 of them had been pregnant, nine were at the postpartum stage, and 107 had a mean lactation time of 1.79 ± 2.01 years. The mean time since the last parturition was 8.12 ± 6.16 years and nine cases were pregnant in the last two months. The subjects’ characteristics are demonstrated in Table 2.

The mean pituitary volume was 329.60 ± 177.08 mm^3^ with mean diameters as follow: craniocaudal: 8.58 ± 7.73 mm, anteroposterior: 6.92 ± 2.95 mm, transverse: 14.10 ± 11.11 mm.

The pituitary volume was in significant correlation with the pituitary diameters (craniocaudal: *r* = 0.53, anteroposterior: *r* = 0.27, transverse: *r* = 0.35, *p* < 0.001 each).

### 3.2. The Correlation Between Pituitary Size and Hormone-Related Factors

The pituitary volume correlated negatively with hormone-related factors. The gravity and parity both had a significant negative effect on pituitary volume (*r* = −0.35, *p* < 0.001, *r* = −0.35, *p* < 0.001, respectively). The use of oral contraceptives and lactation were also in negative correlation with the pituitary volume (*r* = −0.20, *p* = 0.006, *r* = −0.56, *p* < 0.001, respectively).

The craniocaudal diameter was also affected by gravity (*r* = −0.62, *p* < 0.001), parity (*r* = −0.57, *p* < 0.001), intake of contraceptives (*r* = −0.32, *p* < 0.001), and lactation (*r* = −0.70, *p* < 0.001). 

The anteroposterior diameter of the pituitary gland associated significantly with gravity (*r* = −0.19, *p* = 0.009), parity (*r* = −0.20, *p* = 0.007) and lactation (*r* = −0.25, *p* = 0.001), but not with contraceptive intake.

The transverse diameter of the pituitary gland also related negatively with reproductive factors. The correlation between transverse diameter and the evaluated factors were gravity (*r* = −0.15, *p* = 0.04), parity (*r* = −0.17, *p* = 0.02), and lactation (*r* = −0.17, *p* = 0.02), while the intake of contraceptives didn’t correlate with the diameter of the pituitary gland.

### 3.3. Effect of Giving Birth

The mean pituitary volume was 304.30 ± 106.4 mm^3^ in women who had at least a child. This parameter was measured 365.29 ± 160.8 mm^3^ in women during the postpartum time interval and 492.69 ± 517.2 mm^3^ in nullipara women. This difference was statistically significant when comparing three study groups (*p* < 0.001).

The mean anteroposterior diameter was 9.07 ± 1.5 mm in women who had a history of giving birth to a child and 9.36 ± 1.8 mm in women at the postpartum period. Nullipara women had a mean diameter of 9.61 ± 1.8 mm. The analysis revealed a significant difference in the anteroposterior diameter of each of the studied groups (*p* < 0.001). 

The mean craniocaudal diameter was 5.12 ± 2.2 mm in women with parturition history, 5.61 ± 1.3 mm in those at the postpartum stage, and 5.70 ± 14.5 mm in nullipara females.

**Figure 2:**
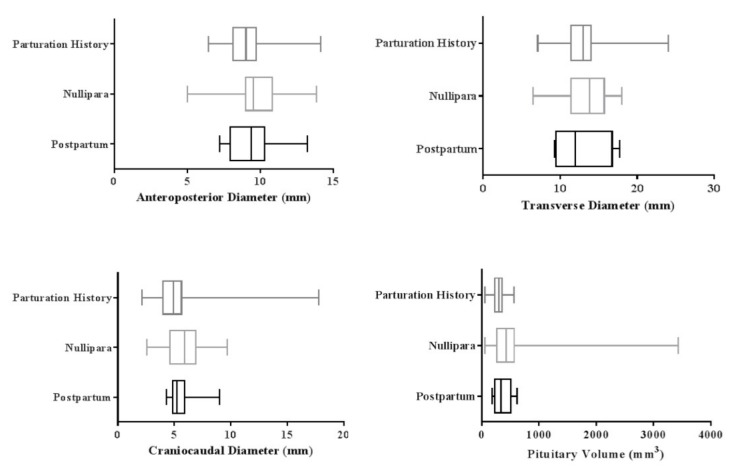
Effect of giving birth on the anteroposterior, craniocaudal, transverse diameter and pituitary volume.

Subjects with a history of parturition had a mean transverse diameter of 12.80 ± 2.9 mm. This parameter was 12.90 ± 3.5 mm in women at the postpartum stage and 13.40 ± 2.8 mm in nullipara women (*p* < 0.001). Figure 1 and 2 illustrates the mean and confidence interval of each of the pituitary diameters in the females at the postpartum period, nullipara, and multipara females.

### 3.4. Effect of Pregnancy

There is a possible effect of pregnancy on pituitary size; therefore, we compared the values in postpartum women with all other subjects. Nine females were at the postpartum stage among the studied subjects. The mean diameters and volume of the gland in the postpartum women compared with the rest of the population is shown in Figure 2. As illustrated, the mean craniocaudal and anteroposterior diameters of the women at the postpartum time was significantly higher than the other women (*p* < 0.001).

**Figure 3:**
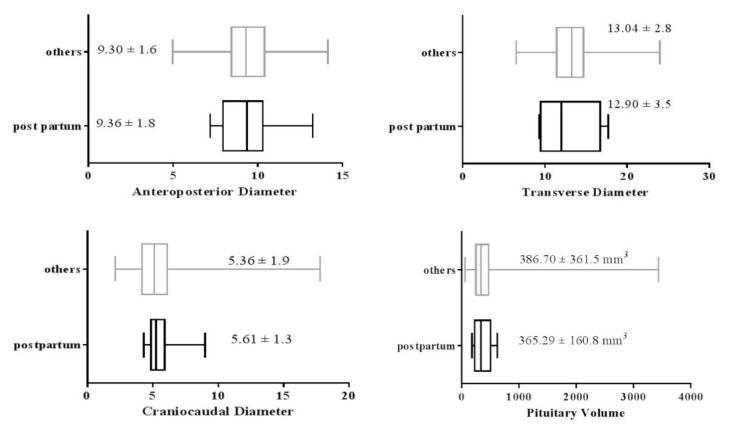
Effect of pregnancy on the anteroposterior, craniocaudal, transverse diameter and pituitary volume

The mean volume and transverse diameter of the women who were recently pregnant did not differ significantly in comparison with non-pregnant women (*p* > 0.05). The mean and confidence interval of each pituitary diameter in the females at the postpartum period compared with other study subjects is demonstrated in Figure 3.

### 3.5. Effect of Oral Contraceptives

**Figure 4:**
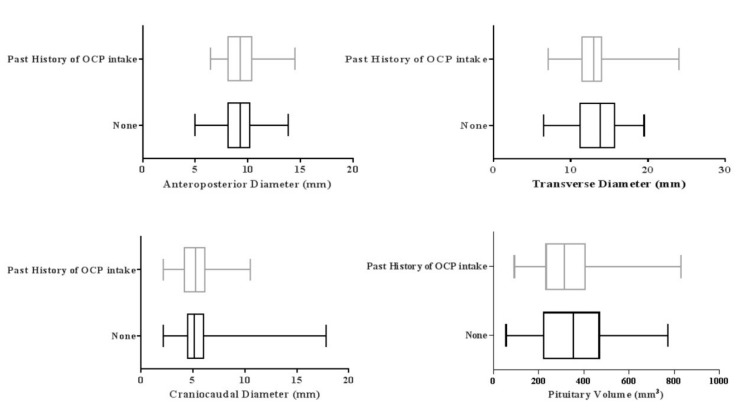
Effect of Oral contraceptives on the anteroposterior, craniocaudal, transverse diameter and pituitary volume.

Fifty-two of the females had a history of the intake of oral contraceptives for 5.66 ± 4.6 months. The pituitary volume was significantly greater in females without a history of the oral contraceptive intake (*p* = 0.05). The mean volume of the pituitary in these females was 326.68 ± 137.2 mm^3^, while this value was 357.74 ± 174.3 mm^3^ in those who did not use oral contraceptives (Figure 4). 

Females who had a history of contraception with oral pills had a mean craniocaudal diameter of 5.37 ± 1.6 mm, and transverse diameter of 13.00 ± 2.8 mm. While insignificant (*p* > 0.05), the values were lower than the mean diameters in females without a history of oral contraception. In females who did not use oral contraceptives, the mean craniocaudal diameter and transverse diameters were 5.40 ± 2.1 mm and 13.26 ± 3.0, respectively.

The mean anteroposterior diameters were 9.37 ± 1.6 mm and 9.19 ± 1.8 mm in females with and without a history of oral contraception, respectively. These also did not differ significantly in two studied groups (*p* > 0.05).

### 3.6. Correction for Age

In multiple regression analysis, age was the most dominant factor with a reverse impact on pituitary volume (*r* = −0.41, *p* < 0.001). After the corrections for age-dependent effects, the correlation between the pituitary volume and reproductive factors remained significant. After correction for the age, the most dominant factors on the pituitary volume was post-parturition and lactation. However, the effect of contraceptives on pituitary volume was found to be insignificant.

The craniocaudal diameter was still associated significantly with reproductive factors after correction for age. Similarly, the correlation between the anteroposterior diameter and the reproductive factors remained as previous. However, none of the factors were related to the transverse diameter. The correlation of the postpartum hemorrhage and time since the last parturition with gland size were also evaluated after correction for age-dependency. The findings revealed a negative association between the time since last parturition and the pituitary volume and craniocaudal diameter: *r* = −0.15, *p* = 0.04 and *r* = −0.34, *p* < 0.001, respectively. These two factors had no linear relation with anteroposterior and transverse diameters. Postpartum hemorrhage also had an inverse association with the pituitary volume and craniocaudal diameter (*r* = −0.18, *p* = 0.01 and *r* = −0.30, *p* < 0.001, respectively), and no relation with anteroposterior and transverse diameters.

## 4. Discussion

### 4.1. Principal Findings

In this prospective population-based study, we evaluated the effect of factors that are related to female hormones on the size of the pituitary gland in females at productive ages. We found a negative effect of ageing on pituitary size. Females’ hormonal factors related to reproduction had a more significant impact on the pituitary volume and craniocaudal diameter. The gravity and parity had a negative impact on the pituitary size, which was an age-independent factor. The years since the last parturition and postpartum hemorrhage both had an inverse age-independent influence on the pituitary volume and craniocaudal diameter. Interestingly, nullipara females had the greatest size of the gland than the other females. Recent pregnancy led to increased craniocaudal and anteroposterior diameters, but not a meaningful change in volume or transverse diameter. The intake of oral contraceptives also had a reductive effect on the volume of the pituitary.

### 4.2. Strengths and Weaknesses of the Study

This study is the first in the literature that was conducted on a large population of females at the reproductive stage to evaluate the size of the pituitary gland. The study population were evaluated based on the factors related to the previous gravity and parity. The values were corrected for the age and females at the postpartum phase were evaluated separately. The method we used for the measurement of pituitary size was accessible and easy to use. We also provided a mean value for each diameter and volume of the gland in females at the reproductive period, which can be used as a control value for future studies.

The major weakness of our findings was that we didn’t follow the participants to evaluate the range of changes in the pituitary diameter of each subject. Another limitation of the study was the low number of females at the postpartum stage. There were only nine females in our study who had recent pregnancies. Hence, the generalization of the findings to the whole female population could not be considered.

### 4.3. Findings in Relation to Other Studies

Imaging modalities have improved the diagnosis of major diseases [12,13]. The previous data in the literature confirmed the inverse effect of ageing on the pituitary size [14,15,16]. Similarly, the pituitary volume decreased as the age increased in our study. The corrections for the age altered our analysis for the transverse diameter. Thus, it should be considered as a main influential factor when measuring the size of the pituitary gland.

In the present study, the longer time since the last parturition diminished the pituitary volume and the craniocaudal diameter; however, Grams et al. couldn’t find any correlations between time-related factors other than age on pituitary size [9].

Similar to Grams et al., parity and intake of oral contraceptives both reduced the pituitary volume in our study.

Earlier studies have described an increased pituitary size during pregnancy followed by the postpartum phase of giving birth [8,10]. Similarly, pregnancy had a significant direct impact on the enlargement of the pituitary gland compared to other normal subjects. However, the nullipara females had greater values than recent pregnant females.

Two former studies have described that the increased pituitary diameters would return to normal size after 1–6 weeks of giving birth [1,17]. The results of our study showed that after giving birth, the pituitary gland reaches a size that is smaller than the size during pregnancy or before it.

There are various and, in some context, divergent data regarding the possible impact of oral contraceptives on the pituitary gland. Some earlier researchers proposed that intake of oral contraceptives stimulates the growth of lactotroph cells of the pituitary, which can lead to enlargement of the gland and even the development of pituitary adenomas [18,19].

On the other hand, our data of 52 females who took oral contraception contradicted the former finding. The use of oral pills diminished each diameter of the pituitary gland, which led to an overall 8.68% reduction in the pituitary volume. Similar findings have been published by Grams et al. in a small sample of 18 females [9].

Abech et al. suggested that hormonal therapy with estrogen induces an enlarged pituitary height in menopausal women [20]. We also found an increased insignificant height (craniocaudal diameter), anteroposterior diameter, and reduced width (transverse diameter) in women who used hormonal contraceptives.

Some previous findings also described the confounding effect of age [16], thyroid anomalies [21,22], and postnatal period [10] on the pituitary size. We corrected the data for age, evaluated the females at the postpartum period separately, and excluded the participants with hyper/hypothyroidism.

### 4.4. Possible Mechanisms

The pituitary gland is the main structure in the human endocrine system. The secretion of gonadotropin hormones, thyroid hormones, and prolactin are regulated by the pituitary and influence the release of pituitary hormones in return [2].

In females, hyperestrogenic states, including pregnancy and the postpartum period, as well as oral contraceptives have shown to increase the pituitary size [20,23,24]. During pregnancy, the level of estrogen increases in the blood, which provokes the proliferation of lactotrophs to 40%. Furthermore, the elevated level of maternal estrogen, diverts more blood supply to the pituitary, which also stimulates the gland overgrowth [2]. These two mechanisms can justify the increased size and volume of the pituitary gland in women during the postpartum period.

On the other hand, the elevated levels of placental hormones suppress the pituitary gonadotrophs, which are located on the lateral sides of the gland [25]. Considering this physiological basis, the transverse diameter of the pituitary was the least affected parameter of the pituitary in our study.

The unexpected result was the higher pituitary volume in nulliparous cases than the multiparous subjects. The occurrence of pituitary hyperplasia could be considered as a potential mechanism for the enlarged size of the pituitary in nullipara females [26]. The idiopathic pituitary hyperplasia can occur due to the overgrowth of various hypophysial cells [27].

Moreover, the use of exogenous estrogen is associated with hyperplasia of lactotrophs, as well as prolactin-secreting adenomas in menopause females [28]. Our observation of the reverse influence of oral contraceptives can be related to the target sample of non-menopause females and the alteration in the pills contents, which contain additional hormones and substances.

### 4.5. Unanswered Questions and Future Research

In the present study, we observed some brand-new findings with insufficient physiological justifications. First, the nulliparous females were found to have the greatest pituitaries when they were compared to multiparous females and those in the postnatal period. The sample population of females in the postpartum period was small in the present study and didn’t contain pregnant females. Therefore, further investigation on a larger sample of pregnant females is required. Second, we couldn’t find a rational reason for the lower size of the pituitary in women who used oral contraceptives, and more research should be applied especially in serum gonadal hormones to find the answer.

## 5. Conclusions

In this study, we found a negative effect of pregnancy, giving birth, and intake of oral contraceptives on the pituitary size. Gravity and parity had the greatest impact on the pituitary volume and craniocaudal diameter. Nullipara females were found to have the largest pituitaries, even larger than the females in the postpartum period.

## Figures and Tables

**Table 1:** Indications for MRI.

Suspicion/Diagnosis	Cases (Number)	Cases (Frequency)
Ataxia	2	1.1
Blurred vision	15	8.3
Dizziness	7	3.8
Dysarthria	4	2.2
Evaluation for ischemia	3	1.6
Headache	92	51.1
Multiple Sclerosis	2	1.1
Nausea	7	3.9
Numbness	19	10.5
Vertigo	29	16.1

**Table 2:** Subjects’ characteristics according to the factors related to reproduction.

Parameter		Mean/Number (percent)
Age	Mean time	33.12 ± 8.67 years
Marital Status	Single	6 (3.3%)
	Married	174 (96.6%)
Pregnancies	Nulligravida	56 (31.1%)
	Unigravida	41 (22.8%)
	Digravida	31 (17.2%)
	Multigravida	52 (28.8%)
Givebirths	Nullipara	65 (36.1%)
	Unipara	41 (22.7%)
	Dipara	52 (28.8%)
	Multipara	22 (12.2%)
Intake of Contraceptives	Cases	52 (28.8%)
	Mean time	5.66 ± 4.6 months
Lactation	Cases	107 (59.4%)
	Mean time	1.79 ± 2.01 years
Postpartum hemorrhage	Cases	40 (22.2%)
Hormone Levels	Cases	64 (30.76%)
	FSH	16.56 ± 7.80 IU/mL
	LH	18.40 ± 11.45 IU/L
	PRL	16.65 ± 8.7 ng/mL
